# Genomic Risk Profiling of Ischemic Stroke: Results of an International Genome-Wide Association Meta-Analysis

**DOI:** 10.1371/journal.pone.0023161

**Published:** 2011-09-21

**Authors:** James F. Meschia, Andrew Singleton, Michael A. Nalls, Stephen S. Rich, Pankaj Sharma, Luigi Ferrucci, Mar Matarin, Dena G. Hernandez, Kerra Pearce, Thomas G. Brott, Robert D. Brown, John Hardy, Bradford B. Worrall

**Affiliations:** 1 Department of Neurology, Mayo Clinic, Jacksonville, Florida, United States of America; 2 Department of Neurology, Mayo Clinic, Rochester, Minnesota, United States of America; 3 Department of Molecular Neuroscience Institute of Neurology, University College London, London, United Kingdom; 4 National Institute on Aging, Bethesda, Maryland, United States of America; 5 Departments of Public Health Sciences and the Center for Public Health Genomics, University of Virginia, Charlottesville, Virginia, United States of America; 6 Imperial College Cerebrovascular Research Unit (ICCRU), Imperial College London and Hammersmith Hospitals, London, United Kingdom; 7 Department of Clinical and Experimental Epilepsy, University College of London Institute of Neurology, London, United Kingdom; 8 University College London Genomics and Institute of Child Health, London, United Kingdom; 9 Departments of Neurology and Public Health Sciences, University of Virginia, Charlottesville, Virginia, United States of America; University of Texas M. D. Anderson Cancer Center, United States of America

## Abstract

**Introduction:**

Familial aggregation of ischemic stroke derives from shared genetic and environmental factors. We present a meta-analysis of genome-wide association scans (GWAS) from 3 cohorts to identify the contribution of common variants to ischemic stroke risk.

**Methods:**

This study involved 1464 ischemic stroke cases and 1932 controls. Cases were genotyped using the Illumina 610 or 660 genotyping arrays; controls, with Illumina HumanHap 550Kv1 or 550Kv3 genotyping arrays. Imputation was performed with the 1000 Genomes European ancestry haplotypes (August 2010 release) as a reference. A total of 5,156,597 single-nucleotide polymorphisms (SNPs) were incorporated into the fixed effects meta-analysis. All SNPs associated with ischemic stroke (P<1×10^−5^) were incorporated into a multivariate risk profile model.

**Results:**

No SNP reached genome-wide significance for ischemic stroke (P<5×10^−8^). Secondary analysis identified a significant cumulative effect for age at onset of stroke (first versus fifth quintile of cumulative profiles based on SNPs associated with late onset, ß = 14.77 [10.85,18.68], P = 5.5×10^−12^), as well as a strong effect showing increased risk across samples with a high propensity for stroke among samples with enriched counts of suggestive risk alleles (P<5×10^−6^). Risk profile scores based only on genomic information offered little incremental prediction.

**Discussion:**

There is little evidence of a common genetic variant contributing to moderate risk of ischemic stroke. Quintiles based on genetic loading of alleles associated with a younger age at onset of ischemic stroke revealed a significant difference in age at onset between those in the upper and lower quintiles. Using common variants from GWAS and imputation, genomic profiling remains inferior to family history of stroke for defining risk. Inclusion of genomic (rare variant) information may be required to improve clinical risk profiling.

## Introduction

Ischemic stroke is known to aggregate in families. The observed familial aggregation is believed to be the result of shared genetic and environmental factors. The true extent of familial aggregation remains unknown, as many individuals who would develop ischemic stroke in later ages may succumb to other morbid conditions, thereby reducing the estimated familial risk. Nonetheless, there are consistent and compelling data that suggest genetic factors play a major role in risk of ischemic stroke.

Ischemic stroke is clinically heterogeneous, with multiple etiologic pathways contributing to risk. There is increasing evidence from genetic studies supporting the hypothesized heterogeneity of ischemic stroke [1]. Many candidate genes have been examined for risk of ischemic stroke, and several Mendelian disorders (*e.g*., CADASIL and Fabry disease) have been associated with stroke risk. Additionally, genome-wide approaches have identified several additional loci associated with stroke risk. The well-documented chromosome 9p21 locus associated with myocardial infarction also has been shown to be a risk factor for large vessel (atherosclerotic) ischemic stroke [2]. The 4q25 locus near the *PITX2* gene that is associated with atrial fibrillation is also associated with cardioembolic stroke [3]. Variants in the *ZFHX3* gene on chromosome 16q22 associate with both atrial fibrillation and cardioembolic stroke [4].

This study involves collaboration among three clinical stroke cohorts comprising two independent datasets that have been subjected to genome-wide association scans. Imputation to greater than 5 million SNPs was performed to permit meta-analysis of association with ischemic stroke and secondary analyses of SNP associations with presumed stroke etiology (subtype) and age at stroke onset. No SNP exhibited genome-wide significant levels of association with ischemic stroke. Risk profiling to identify possible genetic factors associated with ischemic stroke phenotypes identified a potential genetic contribution to ischemic stroke etiology.

## Materials and Methods

### Ethics Statement

All subjects provided written informed consent to participate in stroke genetics research. SWISS and ISGS protocols are approved by the Mayo Clinic Institutional Review Board, Rochester, MN and the BRAINS protocol is approved by the Ethics Committee of Imperial College London & Hammersmith Hospital.

### ISGS/SWISS Dataset

The Ischemic Stroke Genetic Study (ISGS) is a multicenter inception cohort study [5]. Cases were recruited from inpatient stroke services at five United States academic medical centers. Cases are adult men and women over the age of 18 years diagnosed with first-ever ischemic stroke confirmed by a study neurologist on the basis of history, physical examination and CT or MR imaging of the brain. Cases had to be enrolled within 30 days of onset of stroke symptoms. Cases were excluded if they had: a mechanical aortic or mitral valve at the time of the index ischemic stroke, central nervous system vasculitis, or bacterial endocarditis. They were also excluded if they were known to have: cerebral autosomal dominant arteriopathy with subcortical infarcts and leukoencephalopathy (CADASIL), Fabry disease, homocystinuria, mitochondrial encephalopathy with lactic acidosis and stroke-like episodes (MELAS), or sickle cell anemia. Stroke severity at enrollment was assessed using the NIH Stroke Scale and outcomes at 90-days were assessed by telephone using the Barthel Index, Glasgow Outcome Scale, and the modified Rankin scale. Diagnostic evaluation included: head CT (95%) or MRI (83%), electrocardiography (92%), cervical arterial imaging (86%), and echocardiography (74%). Medical records from all cases were centrally reviewed by a vascular neurology committee and assigned ischemic stroke subtype diagnoses according to criteria from the Trial of ORG10172 (TOAST) [6], the Oxfordshire Community Stroke Project [7], and the Baltimore-Washington Young Stroke Study [8]. DNA was donated to the NINDS DNA Repository (Coriell Institute, Camden, NJ) for eligible samples with appropriate written informed consent.

The Siblings with Ischemic Stroke Study (SWISS) is a multicenter affected sibling pair study [9]. Probands with ischemic stroke were enrolled at 66 US medical centers and 4 Canadian medical centers. Probands are adult men and women over the age of 18 years diagnosed with ischemic stroke confirmed by a study neurologist on the basis of history, physical examination and CT or MR imaging of the brain. Probands were required to have a history of at least one living sibling with a history of stroke. Probands were excluded if they had: a mechanical aortic or mitral valve at the time of the index ischemic stroke, central nervous system vasculitis, or bacterial endocarditis. Probands were also excluded if they were known to have: cerebral autosomal dominant arteriopathy with subcortical infarcts and leukoencephalopathy (CADASIL), Fabry disease, homocystinuria, mitochondrial encephalopathy with lactic acidosis and stroke-like episodes (MELAS), or sickle cell anemia. Siblings were enrolled using proband-initiated contact [10] or direct contact when permitted by Institutional Review Boards.

Concordant siblings had their diagnosis of ischemic stroke confirmed by review of medical records by a central vascular neurology committee. Concordant siblings had the same eligibility criteria as probands. Subtype diagnoses were assigned to the index strokes of probands and concordant siblings according to TOAST criteria [6]. Discordant siblings of the proband were confirmed to be stroke-free using the Questionnaire for Verifying Stroke-free Status [11]. A repository of lymphoblastoid cell lines was created and is curated by the Coriell Institute, Camden, NJ.

Readily available US controls were utilized, including stroke-free participants from the Baltimore Longitudinal Study of Aging and the National Institute of Neurological Diseases and Stroke neurologically normal control series taken from the Coriell Cell Repositories. All controls had been previously genotyped and described in detail elsewhere [12].

### Bio-Repository of DNA in Stroke (BRAINS) dataset

BRAINS is an ongoing, multicenter, in-hospital study which recruits consenting acute stroke patients into a highly characterized biobank [13]. All adult (>18 years of age) stroke patients with either ischemic or hemorrhagic pathology were recruited. All patients receive a neurological examination and are required to have either CT or MRI-confirmed lesions. Ischemic stroke subtypes are further sub-classified according to TOAST criteria. All known monogenic causes of stroke are excluded. The BRAINS design has two principal arms. The first arm recruits United Kingdom (UK) European ancestry stroke patients, while the second arm recruits South Asian ancestry stroke patients from multiple sites in the UK and also from sites in India. Neurologically normal control data for the European arm is provided by collaborators at University College London and Cardiff University [14] [15], while control subjects for the South Asian arm are recruited simultaneously as the affected stroke patient and usually is the spouse of the proband. For the purposes of this study, only subjects from the European arm were included.

### Genotyping Quality Control

Both the ISGS/SWISS and the BRAINS genotyping datasets underwent identical quality control procedures. Each case series was genotyped using the Illumina 610 or 660 genotyping arrays, while control series used in the ISGS/SWISS dataset were genotyped using the Illumina HumanHap 550Kv1 or 550Kv3 genotyping arrays. The BRAINS dataset utilized controls genotyped on either the Illumina 610 or 660 genotyping arrays. Genotypes were called using Illumina GenomeStudio software, with all alleles called on the forward strands based on default cluster files provided by Illumina. In addition, all A/T and G/C SNPs were removed prior to merging case and control sample sets, SNPs with discordant minor alleles on the same strand across chips were removed prior to merging datasets as well. Preliminary exclusion criteria per sample included genome-wide SNP call rates <95% and discordance between self-reported gender and sex determined from X chromosome heterozygosity. After merging with control datasets, SNPs were excluded if genotyping success rate <95%, minor allele frequency (MAF) <0.01, Hardy-Weinberg equilibrium (HWE) P<1×10^−4^ in controls and P<1×10^−7^ in cases, nonrandom missingness per haplotype P<1×10^−5^ and missingness in cases compared to controls (from chi-squared test) P<1×10^−5^.

Stroke cases and controls were merged with a subset of samples from HapMap 3 (ASW, CEU, CHB, JPT, TSI and YRI populations) and underwent multidimensional scaling analyses to verify European ancestry for the case-control series. Individuals having estimated principal component vector 1 (PC1) and 2 (PC2) values greater than 3 standard deviations from the combined CEU/TSI means for each vector were excluded as outliers. Evidence of cryptic relatedness was examined using pairwise identical by descent (IBD) estimates. Samples were excluded if they shared greater than a 0.125 proportion of alleles (pi_hat >0.125). After samples were excluded, SNP-based quality control was repeated prior to imputation, with all SNPs passing quality control entering the imputation phase. Basic quality control of genotyped SNP data was carried out using PLINKv1.07 [16]. After quality control was complete, the ISGS/SWISS dataset included 1070 cases and 1488 controls genotyped at 419,170 SNPs and the BRAINS dataset included 400 cases and 444 controls genotyped at 496,742 SNPs. The comparatively lower number of SNPs passing quality control in the ISGS/SWISS dataset is primarily due to issues with merging SNPs across Ilumina arrays, which effectively limited the arrays to the consensus SNPs.

### Imputation

The SNPs passing quality control for the ISGS/SWISS and BRAINS datasets were imputed separately using a two-stage procedure implemented in Markov Chain based haplotyper (MACH; version 1.0.16) [17]. The first stage of imputation generated error and crossover map parameter estimates for the imputation model using a random subset of 200 samples per dataset with over 100 iterations of the initial statistical model. These parameter estimates were used to generate maximum likelihood estimates of allele dosages per SNP from reference haplotypes during the second stage of the imputation. For this study, the August 2010 release of the 1000 Genomes European ancestry haplotypes was utilized as a reference for SNP imputation [18].

### GWAS Statistical Methods

All dataset-specific GWAS summary statistics were generated using logistic regression as implemented in MACH2DAT for binomial phenotypes (*e.g*., ischemic stroke) or linear regression for continuous phenotypes (*e.g*., age at onset of ischemic stroke) as implemented in MACH2QTL [17]. Age at onset was relatively normally distributed in both datasets and did not undergo transformation. Both series of regression models implemented covariates of PC1 and PC2 from multidimensional scaling analyses to adjust for approximate population substructure within each dataset. For each phenotype, a fixed-effects-inverse variance weighted meta-analyses was used (METAL) to combine test statistics across datasets to generate combined P-values for each SNP [19]. Prior to combining P-values, SNPs missing in either study due to post-imputation filtering based on RSQR quality indexes <0.3 or minor allele frequencies <0.01 in either study were removed. Standard errors of the ß coefficients were scaled by the square root of study-specific genomic inflation factor estimates before combining the summary statistics across datasets if the genomic inflation factor was >1. A total of 5,156,597 SNPs (genotyped and imputed) were incorporated into the meta-analysis.

### Risk Profiling

The SNPs chosen for the models were based on meta-analysis of both ISGS/SWISS and BRAINS data. All SNPs with fixed-effects P<1×10^−5^ and appearing in both datasets for each phenotype were incorporated into risk profile modeling. Summary statistics, including effect heterogeneity estimates, for each of these SNPs can be found in Table S1a–S1e. Effect estimates (beta coefficients (ßj) from logistic regression for binomial phenotypes and from linear regression for the continuous age at onset phenotype) from the ISGS/SWISS dataset for these SNPs were used to weight allele counts and estimate risk profiles in the BRAINS dataset. The risk profile (RP), was calculated as follows - for the ‘p’ SNPs, RP = Σjßj_j_*N (j = 1..,p), where ßj_j_ is the parameter estimate for the j^th^ SNP with fixed-effects P<1×10^−5^ in the ISGS/SWISS dataset, and N is the number of risk alleles at the j^th^ SNP (N = 0, 1, 2). The effect estimates from ISGS/SWISS data for the chosen SNPs were then applied to BRAINS data. Risk profile associations for each quintile was quantified using the lowest quintile of risk per population as a reference group in logistic regression models, adjusted for estimates of population substructure (PC1 and PC2 from multi-dimensional scaling) in the BRAINS dataset for all binomial (ischemic stroke) phenotypes. Risk profile associations in the BRAINS dataset for the age at onset phenotype were estimated using linear regression and adjusted for population substructure. Overall risk trends (unstratified models) were evaluated for each population using identical covariates in multivariate regression models.

In the risk profile analysis, age-at-onset was treated as a continuous measure. For this analysis, the quintile groups were based on the distribution of alleles per sample, which is indicative of earlier onset stroke. Therefore the first quintile would be participants possessing the lowest number of alleles associated with early onset of first stroke as the reference population for all analyses, with the fifth quintile (as per Table 1) being participants possessing the highest number of alleles associated with earlier onset of first stroke.

**Table 1:** Risk profile estimates for phenotypes of interest.

			1^st^ Risk Quintile, RG	2^nd^ Risk Quintile	3^rd^ Risk Quintile	4^th^ Risk Quintile	5^th^ Risk Quintile
Binomial Phenotypes*	Trend P-Value	AUC	OR (95% CI)	OR (95% CI)	OR (95% CI)	OR (95% CI)	OR (95% CI)
Ischemic Stroke	4.61E-06	0.605	1	1.64 (1.05, 2.58)	1.81 (1.15, 2.86)	2.25 (1.43, 3.55)	2.75 (1.76, 4.36)
Large Artery	3.98E-10	0.696	1	1.23 (0.53, 2.90)	1.92 (0.90, 4.26)	4.01 (1.95, 8.73)	5.32(2.68, 11.26)
Small Vessel	1.80E-08	0.691	1	0.81 (0.35,1.86)	1.84 (0.86, 4.02)	1.80 (0.89, 3.76)	5.50 (2.70, 11.95)
Quantitative Phenotype	Trend P-Value	Multiple r^2^	Beta (95% CI)	Beta (95% CI)	Beta (95% CI)	Beta (95% CI)	Beta (95% CI)
Age at Onset in years#	1.95E-05	0.0403	0	−9.06 (−12.84, −5.28)	−10.61 (−14.39, −6.83)	−11.10 (−15.12, −7.08)	−14.78 (−18.84, −10.73)

Abbreviations: RG; Reference Group, AUC; area under the curve, OR; Odds Ratio, CI; Confidence Interval.

* Denotes models could not be fit accurately due to only 40 cardioembolic cases, although the overall risk profile trend was significant (Beta = 6.58, Standard error = 1.99, p-value = 0.000936).

# Denotes analysis with reference group as the quintile possessing the fewest alleles associated with earlier onset stroke, as an example, the 5^th^ quintile is the group comprised of participants with the highest cumulative allele dosages associated with earlier onset stroke, mean ages at onset per quintile as follows from 1^st^ to 5^th^ quintiles: 77.51, 69.09, 67.23,66.82, and 63.08 years.

Estimated risk per allele was scaled based on effect estimates from the ISGS/SWISS dataset and fitted to the BRAINS dataset, with nominated SNPs including all SNPs meeting a p-value threshold of 10^−5^ in the meta-analysis specific to each phenotype.

### Post-Hoc Power Calculations

Due to the comparatively small size of this meta-analysis, post-hoc power calculations were carried out. Based on the realistic target of alleles with beta coefficients of roughly 0.3 (odds ratio ∼1.35) in our total series of samples, this study was at 70% power for effect alleles at a frequency of 0.45, 66% power for effect alleles at a frequency of 0.30, and 28% power for effect alleles at a frequency of 0.15, using a P<5×10^−8^ cut-off for significance. The small effect size often associated with common variants is likely the reason for this study failing to identify any genome-wide significant SNP associations. Using a similar modeling scenario for the risk profile analysis, based on the entire BRAINS dataset, we were optimistically powered to detect cumulative risk effects at P<0.01 significance level based on effect sizes at an odds ratio of 1.64 as per the lowest estimate for overall stroke in Table 2. This study surpasses 80% power to detect effects of this size in the risk profiling analysis. Although, this increased power may be an overestimation, as the BRAINS study was used to identify these candidate SNPs in the discovery phase. The low number of tests in the profile scoring analyses helped to alleviate the detrimental effects of multiple testing phenomena on the power of these analyses.

**Table 2:** Descriptive information for GWAS datasets.

	Meta-Analysis			ISGS/SWISS^1^			BRAINS^2^		
Phenotype	Lambda*	Cases	Controls	Lambda*	Cases	Controls	Lambda*	Cases	Controls
AAO	0.993	1462	N/A	1.011	1070	N/A	1.011	392	N/A
CE	0.997	287	1932	1.002	247	1488	1.035	40	444
IS	0.999	1464	1932	1.011	1070	1488	1.064	394	444
LAA	0.989	347	1932	1.010	229	1488	1.047	118	444
SVD	0.995	314	1932	1.014	201	1488	1.030	113	444

1 Age at onset mean = 66.619 years (standard deviation = 13.671), 43% male cohort.

2 Age at onset mean = 68.543 years (standard deviation = 14.001), 53% male cohort.

* Genomic Inflation Factor.

Cohort age at onsets are significantly different (|t| = 2.334, p-value = 0.019)This includes estimates of genomic inflation factor (lambda) and case-control counts for phenotypes of interest.

## Results

**Figure 1:**
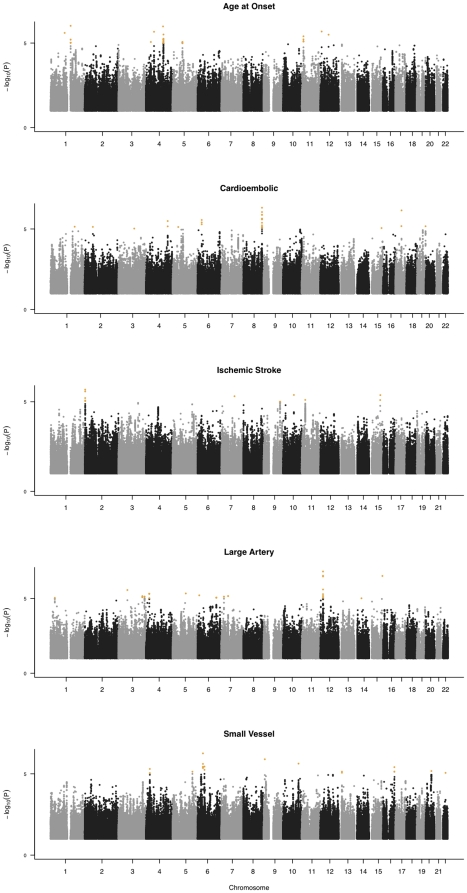
Fixed-effects meta-analysis results for all SNPs passing quality control in both the ISGS/SWISS and BRAINS cohort. Orange points denote loci passed forward to risk profile analyses, with p-values<1E-5 from fixed-effects meta-analyses.

No single SNP (either genotyped or imputed) exhibited genome-wide significant association with ischemic stroke or for any phenotypes analyzed in this study (P<5×10^−8^) (Table 2). Nonetheless, a number of possible candidate regions were identified that approached genome-wide significance. These regions include loci that were incorporated into the risk profile analyses, as they had P<1×10^−5^ in fixed-effects meta-analysis (Figure 1). The implicated chromosome 9p21 locus associated with myocardial infarction, the 4q25 locus near the *PITX2* and variants in the *ZFHX3* were included in our meta-analysis and summary statistics for these implicated loci may be found in the Table S2, although the small effect sizes in original reports from studies with larger sample sizes caused detection of these effects on a genome-wide scale to be impossible.

The risk profile analyses demonstrate significant trends of cumulative genetic effects associated with risk of ischemic stroke and presumed etiology (TOAST subtypes). The association between risk profile SNPs and ischemic stroke age at onset is significant (P = 1.95×10^−5^) after Bonferroni correction for 5 tests and the risk profile accounts for ∼4% of the variation in the phenotype. This association persists after adjustment for stroke subtypes (1.71×10^−5^). The surprisingly strong trends for associations with ischemic stroke and presumed etiology suggest genetic effects that should be detected by larger meta-analyses. There are markedly significant risk increases across more common stroke subtypes (OR [95% confidence interval] as shown in Table 1: ischemic stroke −2.75 [1.76, 4.36], large artery −5.32 [2.68,11.26] and small vessel −5.50 [2.70, 11.95]). There is also a large effect contrasting the first and fifth quintiles of age at onset (ß = −14.77 [−10.85, −18.68], P = 5.54×10^−12^), suggesting a strong skewing of effect towards genetic variants with a high propensity for late onset stroke. When adding additional covariates of stroke subtype into the age at onset model, the overall trend is still highly. The risk profile scorings provide additional suggestive evidence of genetic components in the etiology of stroke. Nevertheless, all area under the curve (AUC) estimates were below 0.7, suggesting little incremental clinical utility of SNP genotype information at this stage, assuming AUC >0.8 is often the criterion for clinical utility.

## Discussion

This genome-wide association study follows a previous published, but low-powered, genome-wide association study that involved 278 patients and 275 controls [20]. The current study has a substantial increase in statistical power, accumulating a sample set of 1464 cases and 1932 controls. Notwithstanding this increase in statistical power, no single SNP reached genome-wide levels of significance for association with ischemic stroke or associated secondary phenotypes. This failure to identify a locus for ischemic stroke with this sample size is consistent with the results of the similarly sized CHARGE consortium, whose initial finding of a locus on chromosome 12 has yet to independently replicate [21] [12]. Meta-analytical techniques applied to substantially larger data sets will be necessary to reliably identify risk loci for ischemic stroke with common variants from GWAS and imputation.

We identified a significant difference that survived correction for multiple testing between the highest quintile and lowest quintile of allele dosages contributing to age at onset of stroke (compared with the lowest quintile), this revealed a 14.78 (95% CI −18.84, −10.73) year difference in age at onset between these two groups (P = 2.45×10^−11^). In the SWISS data, a significant correlation has previously been reported between age at onset for probands with ischemic stroke and age at onset in their ischemic stroke-affected siblings [22]. About 50% of variability in age at onset in a proband could be accounted for by age in an affected sibling. This correlation was likely the result of genetic factors, shared environmental factors and, possibly, to ascertainment bias. Resequencing studies with longitudinal follow-up and detailed environmental exposure data will help researchers delve further into etiological effects influencing age at first stroke, as rare variants and environmental factors may have greater influence on this phenotype than the common variants reported here. Age at stroke is a precise clinical phenotype because stroke by definition is a paroxysmal disorder. In this sense, disease onset is a more precisely defined phenotype in patients with ischemic stroke than it is for patients with chronic neurodegenerative diseases such as Parkinson's or Alzheimer's disease, that are characterized by progression from preclinical to clinical levels over months or years. The parameter of age at onset of ischemic stroke does have some limitations as a biological phenotype as a number of ischemic strokes are “silent”, without symptoms or gross clinical signs [23]. Further, some ischemic strokes generate symptoms, but fail to rise to clinical attention [24].

The observed significant relationships in genomic risk profile between the highest risk quintile and major ischemic stroke subtypes (large vessel and small vessel etiology) is consistent with the known association between family history of stroke and large vessel and small vessel ischemic stroke [25]. However, even though the risk profiling was highly significant for phenotypes like large vessel stroke, risk profiling provided little incremental gain in prediction of ischemic stroke. For large vessel ischemic stroke, profiling explained only 4% of the variance in risk. Our work suggests that genomic profiling using the current SNP technology is unlikely to be a clinically useful way of staging stroke risk as there is no substantial gain over simply including family history of stroke status [26]. However, it may be possible to make incremental improvements in clinical risk profiling by incorporating genomic (rare variant) as well as other “omic” information in the future.

## Supporting Information

Table S1 Single nucleotide polymorphisms with the strongest associations for srtoke.
(DOC)

Table S2 This table shows the association between previously identified stroke loci and related stroke phenotypes in this study (ischemic stroke overall and cardioembolic stroke).
(DOC)
