# Supplementary material for: Genomic Risk Profiling of Ischemic Stroke: Results of an International Genome-Wide Association Meta-Analysis
Source: PLoS One. 2011 Sep 21;6(9):e23161. doi: 10.1371/journal.pone.0023161 (PMC3177829; doi:10.1371/journal.pone.0023161)
Supplement: Table S2 — This table shows the association between previously identified stroke loci and related stroke phenotypes in this study (ischemic stroke overall and cardioembolic stroke). (DOC) [file pone.0023161.s002.doc]

Supplementary Table S2.
