# Supplementary material for: Genomic Risk Profiling of Ischemic Stroke: Results of an International Genome-Wide Association Meta-Analysis
Source: PLoS One. 2011 Sep 21;6(9):e23161. doi: 10.1371/journal.pone.0023161 (PMC3177829; doi:10.1371/journal.pone.0023161)
Supplement: Supplementary file 2 [file pone.0023161.s001.doc]

Supplementary Table S1a: Age At Onset

SNP	Allele	Allele2	Freq1	FreqSE	MinFreq	MaxFreq	Effect	StdErr	Pvalue	Direction.ISGSthenBRAINS	Het.ChiSq	Het.df	Het.Pvalue
chr4:126324873	g	g	0.0759	0.001	0.0743	0.0765	-4.1473	0.9375	9.71E-06	--	1.809	1	0.1787
chr4:126335361	a	a	0.074	8.00E-04	0.0735	0.0752	-4.1659	0.9361	8.57E-06	--	1.965	1	0.161
chr4:126336246	a	a	0.0739	8.00E-04	0.0734	0.0752	-4.1585	0.9353	8.74E-06	--	1.982	1	0.1592
chr4:126340479	g	g	0.0725	0.0012	0.0718	0.0745	-4.2746	0.9423	5.73E-06	--	1.834	1	0.1757
chr4:126340797	a	a	0.073	0.0011	0.0723	0.0748	-4.2378	0.9387	6.35E-06	--	1.466	1	0.2259
chr4:126342638	a	a	0.0728	0.0011	0.0721	0.0746	-4.2402	0.9379	6.16E-06	--	1.429	1	0.2319
chr4:126342673	a	a	0.0728	0.0011	0.0721	0.0746	-4.2409	0.9379	6.14E-06	--	1.428	1	0.2321
chr4:126344103	g	g	0.0728	0.0011	0.0721	0.0746	-4.2396	0.9379	6.18E-06	--	1.427	1	0.2323
chr4:126344137	g	g	0.0728	0.0011	0.0721	0.0746	-4.2396	0.9379	6.18E-06	--	1.427	1	0.2323
chr4:126344266	a	a	0.0729	0.0011	0.0722	0.0746	-4.2393	0.9379	6.19E-06	--	1.426	1	0.2324
chr4:126344643	a	a	0.0729	0.0011	0.0722	0.0746	-4.2393	0.9379	6.19E-06	--	1.426	1	0.2324
chr4:126344730	g	g	0.0729	0.0011	0.0722	0.0746	-4.2393	0.9379	6.19E-06	--	1.426	1	0.2324
chr4:126344897	a	a	0.0729	0.0011	0.0722	0.0746	-4.2393	0.9379	6.19E-06	--	1.426	1	0.2324
chr4:126345106	a	a	0.0729	0.0011	0.0722	0.0746	-4.2393	0.9379	6.19E-06	--	1.426	1	0.2324
chr4:126345124	c	c	0.0729	0.0011	0.0722	0.0746	-4.2386	0.9379	6.21E-06	--	1.427	1	0.2323
chr4:126345153	a	a	0.0729	0.0011	0.0722	0.0746	-4.2383	0.9379	6.22E-06	--	1.426	1	0.2324
chr4:126345479	a	a	0.0729	0.0011	0.0722	0.0746	-4.2383	0.9379	6.22E-06	--	1.426	1	0.2324
chr4:126345483	g	g	0.0729	0.0011	0.0722	0.0746	-4.2389	0.9378	6.18E-06	--	1.427	1	0.2322
chr4:126345500	a	a	0.0729	0.0011	0.0722	0.0746	-4.2386	0.9378	6.19E-06	--	1.426	1	0.2324
chr4:126345904	g	g	0.0729	0.0011	0.0722	0.0746	-4.2384	0.9378	6.20E-06	--	1.425	1	0.2326
chr4:126346501	a	a	0.0729	0.001	0.0723	0.0746	-4.2398	0.9378	6.15E-06	--	1.423	1	0.233
chr4:126347671	g	g	0.0729	0.001	0.0723	0.0746	-4.241	0.9378	6.12E-06	--	1.419	1	0.2336
chr4:126347708	a	a	0.0729	0.001	0.0723	0.0746	-4.2407	0.9378	6.13E-06	--	1.418	1	0.2337
chr4:126348007	a	a	0.0729	0.001	0.0723	0.0746	-4.2414	0.9378	6.10E-06	--	1.417	1	0.2339
chr4:126348017	g	g	0.0729	0.001	0.0723	0.0746	-4.2414	0.9378	6.10E-06	--	1.417	1	0.2339
chr4:126348762	c	c	0.073	0.001	0.0724	0.0746	-4.2426	0.9378	6.07E-06	--	1.413	1	0.2345
chr4:126348812	g	g	0.073	0.001	0.0724	0.0746	-4.2431	0.9378	6.05E-06	--	1.411	1	0.2349
chr4:126348946	a	a	0.073	0.001	0.0724	0.0746	-4.2431	0.9378	6.05E-06	--	1.411	1	0.2349
chr4:126348977	c	c	0.073	0.001	0.0724	0.0746	-4.2435	0.9378	6.04E-06	--	1.409	1	0.2352
chr4:126349574	c	c	0.073	0.001	0.0724	0.0746	-4.244	0.9378	6.03E-06	--	1.407	1	0.2356
chr4:126349641	a	a	0.0731	9.00E-04	0.0725	0.0746	-4.244	0.9378	6.03E-06	--	1.407	1	0.2356
chr4:126349713	a	a	0.0731	9.00E-04	0.0725	0.0746	-4.2447	0.9378	6.00E-06	--	1.406	1	0.2358
chr4:126352213	a	a	0.0731	9.00E-04	0.0725	0.0746	-4.2452	0.9378	5.99E-06	--	1.403	1	0.2362
chr4:126353266	a	a	0.0731	9.00E-04	0.0725	0.0746	-4.2452	0.9378	5.99E-06	--	1.403	1	0.2362
chr4:126353458	a	a	0.0731	9.00E-04	0.0725	0.0746	-4.2456	0.9378	5.98E-06	--	1.401	1	0.2365
chr4:126353758	g	g	0.0731	9.00E-04	0.0725	0.0746	-4.2456	0.9378	5.98E-06	--	1.401	1	0.2365
chr4:126354121	c	c	0.0731	9.00E-04	0.0725	0.0746	-4.2461	0.9378	5.96E-06	--	1.399	1	0.2369
chr4:126357219	g	g	0.0732	9.00E-04	0.0727	0.0747	-4.2492	0.9378	5.87E-06	--	1.403	1	0.2362
rs10015153	g	g	0.0794	6.00E-04	0.0785	0.0798	-4.3566	0.9343	3.11E-06	--	1.746	1	0.1863
rs10032994	a	a	0.1038	0.0032	0.0986	0.1058	-5.5383	1.1339	1.04E-06	--	1.108	1	0.2926
rs10061668	c	c	0.2042	0.0051	0.2012	0.213	-2.7918	0.628	8.78E-06	--	14.817	1	0.0001185
rs10079381	c	c	0.2044	0.0051	0.2014	0.2131	-2.7907	0.6287	9.04E-06	--	14.849	1	0.0001165
rs1013587	c	c	0.3658	0.0016	0.3649	0.3686	2.3217	0.5246	9.63E-06	++	0.66	1	0.4166
rs10715778	c	c	0.0729	0.0011	0.0722	0.0746	-4.2386	0.9378	6.19E-06	--	1.426	1	0.2324
rs10900374	c	c	0.662	0.0027	0.6604	0.6666	-2.5287	0.56	6.32E-06	--	1.623	1	0.2027
rs11021961	a	a	0.2177	0.0026	0.2162	0.2222	-2.8285	0.6339	8.11E-06	--	1.272	1	0.2593
rs11164428	g	g	0.4463	0.0076	0.4419	0.4595	-2.5939	0.5509	2.50E-06	--	2.725	1	0.09882
rs11240065	a	a	0.8852	0.0011	0.8845	0.8869	-5.5504	1.1327	9.57E-07	--	0.279	1	0.5974
rs12046574	a	a	0.3657	0.0014	0.3649	0.3682	2.33	0.5264	9.60E-06	++	0.652	1	0.4193
rs12504677	c	c	0.1108	0.0027	0.1058	0.1123	-5.0789	1.1417	8.65E-06	--	2.186	1	0.1392
rs1344454	a	a	0.5641	0.0038	0.5617	0.5703	2.4705	0.5205	2.08E-06	++	1.223	1	0.2688
rs17009284	a	a	0.0734	0.001	0.0728	0.075	-4.2244	0.9384	6.74E-06	--	1.51	1	0.2192
rs17009293	a	a	0.0733	0.001	0.0727	0.0749	-4.2282	0.9389	6.68E-06	--	1.499	1	0.2209
rs17009295	g	g	0.0732	0.001	0.0726	0.0749	-4.2288	0.9389	6.66E-06	--	1.493	1	0.2218
rs17160321	a	a	0.3658	0.0016	0.3649	0.3687	2.3179	0.5238	9.65E-06	++	0.664	1	0.4151
rs17160322	c	c	0.3658	0.0016	0.3649	0.3687	2.3179	0.5238	9.65E-06	++	0.664	1	0.4151
rs17160327	a	a	0.3658	0.0016	0.3649	0.3686	2.3193	0.524	9.58E-06	++	0.662	1	0.416
rs17608369	g	g	0.053	0.0024	0.0514	0.0566	-5.9724	1.2822	3.19E-06	--	1.631	1	0.2016
rs1845807	c	c	0.6618	0.0027	0.6602	0.6664	-2.5312	0.5601	6.22E-06	--	1.613	1	0.2041
rs2112298	a	a	0.2047	0.005	0.2018	0.2132	-2.7881	0.6309	9.91E-06	--	14.889	1	0.0001141
rs2390774	a	a	0.0759	0.001	0.0743	0.0765	-4.1458	0.9375	9.78E-06	--	1.811	1	0.1784

Supplmentary Table S1b: Ischemic Stroke

SNP	Allele	Allele2	Freq1	FreqSE	MinFreq	MaxFreq	Effect	StdErr	Pvalue	Direction.ISGSthenBRAINS	Het.ChiSq	Het.df	Het.Pvalue
chr2:3058370	g	g	0.7522	0.0095	0.7472	0.7702	-0.2645	0.0596	9.09E-06	--	3.015	1	0.08249
rs10169682	g	g	0.3821	0.0017	0.3812	0.3852	-0.2609	0.0555	2.63E-06	--	0.052	1	0.8199
rs10983013	g	g	0.9116	0.0054	0.9084	0.9206	-0.5033	0.1139	9.90E-06	--	4.318	1	0.03772
rs11028255	g	g	0.5198	0.0027	0.5152	0.5214	-0.2671	0.0598	7.85E-06	--	0.673	1	0.412
rs12714371	a	a	0.3701	0.0014	0.3693	0.3727	-0.2501	0.0563	9.03E-06	--	0.005	1	0.9461
rs1453822	a	a	0.3382	6.00E-04	0.3379	0.3393	-0.2593	0.0574	6.18E-06	--	0.028	1	0.866
rs17315707	c	c	0.9048	0.0016	0.9039	0.9078	0.4509	0.101	8.02E-06	++	0.464	1	0.4959
rs1865020	a	a	0.3419	0.0054	0.3324	0.3449	0.2415	0.0525	4.15E-06	++	0.002	1	0.9608
rs43061	a	a	0.3905	9.00E-04	0.39	0.3921	-0.235	0.0514	4.90E-06	--	2.114	1	0.146
rs4778663	c	c	0.6289	0.0044	0.621	0.6313	-0.2382	0.0518	4.24E-06	--	0.027	1	0.8694
rs4854189	a	a	0.363	1.00E-04	0.3629	0.3632	-0.2678	0.0565	2.09E-06	--	0.001	1	0.9702
rs7371392	a	a	0.6478	0.0081	0.6434	0.6628	-0.2443	0.0549	8.49E-06	--	1.341	1	0.2469
rs7467015	g	g	0.7479	0.005	0.7451	0.7569	-0.2605	0.0589	9.88E-06	--	1.39	1	0.2384
rs9408887	a	a	0.7554	0.0047	0.7528	0.7638	-0.2566	0.058	9.78E-06	--	1.419	1	0.2335

Supplmentary Table S1c: Cardioembolic

SNP	Allele	Allele2	Freq1	FreqSE	MinFreq	MaxFreq	Effect	StdErr	Pvalue	Direction.ISGSthenBRAINS	Het.ChiSq	Het.df	Het.Pvalue	CHR	BP
chr1:173691052	a	a	0.9243	0.0011	0.9221	0.9248	-1.1338	0.2527	7.24E-06	--	2.015	1	0.1557	1	173691052
chr15:88991654	a	a	0.8291	3.20E-03	0.8276	0.8361	1.0766	0.2422	8.80E-06	++	2.032	1	0.154	15	88991654
chr17:43049979	c	c	0.9063	1.90E-03	0.9054	0.9104	-0.8713	0.1934	6.66E-06	--	0.116	1	0.7334	17	43049979
chr17:44355992	a	a	0.941	0.0067	0.9245	0.9437	-1.0131	0.2042	7.02E-07	--	2.231	1	0.1352	17	44355992
chr19:62265183	a	a	0.8499	0.0045	0.8478	0.8594	-0.6445	0.1432	6.79E-06	--	1.514	1	0.2185	19	62265183
chr3:113689765	g	g	0.8075	0.0029	0.8015	0.8089	-0.7379	0.1666	9.43E-06	--	1.052	1	0.3051	3	113689765
chr5:40532016	c	c	0.9288	5.00E-04	0.9275	0.929	-1.0551	0.2357	7.57E-06	--	2.516	1	0.1127	5	40532016
chr6:29782679	c	c	0.9472	2.00E-04	0.9471	0.9476	-0.7971	0.1749	5.14E-06	--	1.766	1	0.1839	6	29782679
chr6:29787887	a	a	0.947	3.00E-04	0.9469	0.9476	-0.8068	0.1748	3.91E-06	--	1.934	1	0.1643	6	29787887
chr6:29789140	g	g	0.947	3.00E-04	0.9468	0.9476	-0.8091	0.1726	2.76E-06	--	1.991	1	0.1582	6	29789140
chr8:133828500	a	a	0.869	0.0058	0.867	0.8856	-0.5477	0.1227	8.10E-06	--	0.669	1	0.4135	8	133828500
chr8:133831026	a	a	0.8689	0.0058	0.8669	0.8855	-0.5475	0.1227	8.17E-06	--	0.677	1	0.4106	8	133831026
chr8:133831077	a	a	0.8632	0.0059	0.8611	0.8798	-0.5438	0.1217	7.91E-06	--	0.917	1	0.3383	8	133831077
chr8:133862452	a	a	0.8656	0.0053	0.8636	0.88	-0.6239	0.1239	4.80E-07	--	0.082	1	0.7753	8	133862452
chr8:133945335	a	a	0.8802	0.0065	0.8778	0.8978	-0.6137	0.1249	8.87E-07	--	0.084	1	0.7725	8	133945335
chr8:133990196	g	g	0.8988	0.0043	0.8971	0.9096	-0.6439	0.133	1.29E-06	--	0.043	1	0.8365	8	133990196
chr8:133998320	g	g	0.8983	0.0041	0.8967	0.9086	-0.6428	0.133	1.35E-06	--	0.042	1	0.8385	8	133998320
chr8:134044365	a	a	0.8924	0.0037	0.8909	0.9017	-0.5943	0.1291	4.12E-06	--	0.004	1	0.9487	8	134044365
rs1963138	c	c	0.9017	0.0038	0.9001	0.9107	-0.6342	0.1401	5.97E-06	--	0.084	1	0.7721	8	134039527
rs2433057	a	a	0.868	0.0062	0.8658	0.8854	-0.5486	0.1234	8.77E-06	--	0.33	1	0.5656	8	133837840
rs28540916	a	a	0.8961	0.0044	0.8944	0.9072	-0.6107	0.1293	2.34E-06	--	0.052	1	0.8198	8	134028581
rs4482740	c	c	0.6503	0.0025	0.6494	0.6572	0.476	0.1022	3.23E-06	++	0.397	1	0.5287	4	155665755
rs4736614	a	a	0.896	0.0042	0.8943	0.9066	-0.6114	0.1293	2.27E-06	--	0.042	1	0.8384	8	134030193
rs6834179	a	a	0.6444	0.0013	0.6408	0.6449	0.5111	0.1137	6.91E-06	++	0.591	1	0.4421	4	155659900
rs6988193	a	a	0.8885	4.00E-04	0.8876	0.8887	-0.5837	0.1301	7.20E-06	--	0.032	1	0.859	8	134049633
rs7015805	a	a	0.8957	0.0036	0.8942	0.9045	-0.6102	0.1298	2.57E-06	--	0.227	1	0.6336	8	134035118
rs7577730	a	a	0.4518	0.0034	0.4504	0.46	0.48	0.1071	7.42E-06	++	0.162	1	0.6878	2	59059458
rs9643296	a	a	0.8958	0.0038	0.8942	0.9052	-0.611	0.1298	2.49E-06	--	0.225	1	0.6356	8	134035530

Supplmentary Table S1d: Large Artery Atherosclerosis

SNP	Allele	Allele2	Freq1	FreqSE	MinFreq	MaxFreq	Effect	StdErr	Pvalue	Direction.ISGSthenBRAINS	Het.ChiSq	Het.df	Het.Pvalue	CHR	BP
chr12:19061620	c	c	0.9393	0.0044	0.9368	0.9471	-0.9938	0.1948	3.37E-07	--	0.62	1	0.431	12	19061620
chr12:19062674	a	a	0.9358	0.0046	0.9332	0.944	-0.9713	0.1896	3.01E-07	--	0.576	1	0.4479	12	19062674
chr12:19073554	c	c	0.8771	0.0036	0.8749	0.8829	-0.6044	0.1354	7.98E-06	--	1.557	1	0.2121	12	19073554
chr1:30766518	a	a	0.8066	0.0028	0.8024	0.8085	0.5559	0.1253	9.07E-06	++	0.618	1	0.4318	1	30766518
chr14:50308166	a	a	0.9423	0.0014	0.9401	0.9432	-0.8134	0.1839	9.71E-06	--	0.182	1	0.6694	14	50308166
chr15:94763682	c	c	0.8919	0.0011	0.8904	0.8927	-0.981	0.1918	3.12E-07	--	0	1	1	15	94763682
chr3:173425605	a	a	0.7688	0.0067	0.7594	0.7736	0.497	0.1112	7.87E-06	++	0.107	1	0.7434	3	173425605
chr3:189193766	a	a	0.0617	0.004	0.059	0.0676	0.6863	0.1535	7.77E-06	++	0.018	1	0.8945	3	189193766
chr4:24362816	g	g	0.8519	0.0054	0.8441	0.8556	-0.4967	0.1086	4.83E-06	--	0.758	1	0.3839	4	24362816
chr5:96005511	g	g	0.8681	0.0039	0.8656	0.8741	-0.7835	0.171	4.61E-06	--	0.009	1	0.9239	5	96005511
chr7:19019258	c	c	0.8484	0.001	0.8477	0.85	-0.5006	0.1118	7.55E-06	--	1.869	1	0.1716	7	19019258
rs10021418	a	a	0.8521	0.0055	0.8442	0.8559	-0.4958	0.1085	4.84E-06	--	0.779	1	0.3775	4	24359180
rs1018298	a	a	0.8348	0.0037	0.8325	0.8408	-0.503	0.1117	6.64E-06	--	0.622	1	0.4304	12	19074039
rs10258912	a	a	0.6968	0.0092	0.6907	0.7106	-0.4027	0.0895	6.88E-06	--	0.333	1	0.5641	7	48688952
rs11044389	c	c	0.843	0.0053	0.8396	0.8511	-0.496	0.1053	2.50E-06	--	0.821	1	0.3648	12	19059848
rs11044400	g	g	0.8115	0.0036	0.8091	0.8169	-0.5842	0.1114	1.57E-07	--	0.222	1	0.6374	12	19077314
rs11712833	c	c	0.771	0.007	0.7612	0.776	0.4964	0.1112	8.08E-06	++	0.074	1	0.7855	3	173431902
rs12194586	g	g	0.9018	0.0031	0.9	0.907	-0.5557	0.125	8.82E-06	--	0.063	1	0.8025	6	134664636
rs12197956	a	a	0.9017	0.003	0.8999	0.9068	-0.5557	0.125	8.82E-06	--	0.063	1	0.8025	6	134662764
rs12204317	a	a	0.9017	0.003	0.8999	0.9068	-0.5557	0.125	8.82E-06	--	0.063	1	0.8025	6	134662908
rs12505599	g	g	0.852	0.0054	0.8442	0.8557	-0.4955	0.1085	4.91E-06	--	0.771	1	0.3798	4	24360032
rs13438050	g	g	0.6968	0.0092	0.6907	0.7106	-0.4027	0.0895	6.88E-06	--	0.333	1	0.5641	7	48688602
rs17507623	c	c	0.8789	0.0053	0.8754	0.8869	-0.8582	0.1898	6.18E-06	--	2.377	1	0.1231	6	11509505
rs17621680	a	a	0.8541	0.0059	0.8454	0.8581	-0.4885	0.1103	9.51E-06	--	0.801	1	0.3706	4	24358633
rs2107595	c	c	0.8465	3.00E-04	0.846	0.8467	-0.4968	0.111	7.68E-06	--	1.835	1	0.1755	7	19015913
rs34718992	a	a	0.7712	0.0069	0.7615	0.7761	0.496	0.1112	8.19E-06	++	0.076	1	0.7822	3	173438723
rs34770855	a	a	0.7682	0.0067	0.7589	0.773	0.5	0.1112	6.94E-06	++	0.116	1	0.7337	3	173422999
rs35198260	a	a	0.0617	0.0039	0.0591	0.0676	0.6877	0.1535	7.45E-06	++	0.019	1	0.8897	3	189200833
rs35898760	g	g	0.771	0.007	0.7613	0.776	0.496	0.1112	8.19E-06	++	0.076	1	0.7822	3	173432875
rs6445051	a	a	0.7711	0.007	0.7614	0.7761	0.496	0.1112	8.19E-06	++	0.076	1	0.7822	3	173436792
rs6851578	g	g	0.852	0.0054	0.8441	0.8557	-0.4958	0.1085	4.84E-06	--	0.779	1	0.3775	4	24359161
rs7431862	c	c	0.9049	0.0035	0.9025	0.91	-1.0016	0.2136	2.73E-06	--	0.469	1	0.4936	3	65225172
rs7763911	a	a	0.9017	0.003	0.8999	0.9068	-0.5557	0.125	8.82E-06	--	0.063	1	0.8025	6	134662800
rs7973505	a	a	0.8775	0.0025	0.8759	0.8813	-0.6834	0.1499	5.13E-06	--	0.015	1	0.9036	12	19055571
rs7979074	c	c	0.8369	0.0042	0.8342	0.8435	-0.4576	0.1032	9.31E-06	--	0.677	1	0.4107	12	19060795
rs9493891	c	c	0.9018	0.0031	0.9	0.907	-0.5557	0.125	8.82E-06	--	0.063	1	0.8025	6	134665533

Supplmentary Table S1e: Small Vessel Disease															
SNP	Allele	Allele2	Freq1	FreqSE	MinFreq	MaxFreq	Effect	StdErr	Pvalue	Direction.ISGSthenBRAINS	Het.ChiSq	Het.df	Het.Pvalue	CHR	BP
chr13:24209694	c	c	0.9337	0.0025	0.9322	0.9379	-0.8758	0.1954	7.35E-06	--	5.691	1	0.01705	13	24209694
chr13:24414775	g	g	0.9371	0.0023	0.9336	0.9386	-1.0072	0.2264	8.61E-06	--	0.141	1	0.7075	13	24414775
chr22:33708256	a	a	0.8973	0.0031	0.895	0.9016	-0.6381	0.1435	8.74E-06	--	0.004	1	0.9472	22	33708256
chr6:39319803	c	c	0.9461	9.00E-04	0.9447	0.9467	-0.8735	0.189	3.80E-06	--	0.642	1	0.423	6	39319803
chr6:39337399	g	g	0.9316	0.0024	0.9301	0.9355	-0.7108	0.1547	4.32E-06	--	0.058	1	0.8096	6	39337399
chr6:39337721	a	a	0.9314	0.0025	0.9299	0.9354	-0.7067	0.1539	4.39E-06	--	0.054	1	0.8156	6	39337721
chr6:39364964	a	a	0.9398	0.0011	0.9391	0.9416	-0.7909	0.1679	2.46E-06	--	0.262	1	0.6085	6	39364964
chr9:9173012	g	g	0.9347	0.0066	0.9254	0.9394	-0.8087	0.1669	1.27E-06	--	0.012	1	0.9146	9	9173012
rs10059999	g	g	0.9191	0.0057	0.9091	0.9224	-0.6346	0.1416	7.41E-06	--	0.961	1	0.327	5	144373578
rs10509906	c	c	0.29	0.0072	0.2788	0.2946	0.5085	0.1077	2.36E-06	++	0.097	1	0.7552	10	111747664
rs11640777	g	g	0.7157	0.0055	0.7119	0.7238	-0.4249	0.0949	7.49E-06	--	3.623	1	0.057	16	82119714
rs12503203	a	a	0.4807	0.0023	0.4792	0.4841	-0.3997	0.0902	9.33E-06	--	2.377	1	0.1231	4	28433398
rs16868846	c	c	0.9461	0.001	0.9447	0.9468	-0.878	0.1897	3.71E-06	--	0.642	1	0.4232	6	39315536
rs2815063	g	g	0.8968	0.0022	0.8954	0.9003	-0.6924	0.1383	5.55E-07	--	0.666	1	0.4145	6	39370513
rs4284623	a	a	0.7673	0.0027	0.7653	0.7709	-0.4773	0.1064	7.29E-06	--	0.879	1	0.3484	16	82113782
rs4479690	a	a	0.3729	0.0021	0.3698	0.3744	-0.4543	0.0995	5.02E-06	--	0.159	1	0.69	4	28436972
rs489184	a	a	0.5495	0.0092	0.5366	0.5561	0.4248	0.0917	3.57E-06	++	0.494	1	0.4823	6	49855421
rs527461	a	a	0.5506	0.0101	0.5367	0.5579	0.415	0.0915	5.70E-06	++	0.35	1	0.5542	6	49833300
rs6844183	a	a	0.4808	0.0022	0.4793	0.484	-0.4003	0.0902	9.03E-06	--	2.409	1	0.1207	4	28430104
rs7187172	a	a	0.7207	0.004	0.718	0.7265	-0.438	0.0949	3.90E-06	--	3.679	1	0.05511	16	82119367
rs8125304	a	a	0.9165	0.002	0.9152	0.9196	-0.6695	0.1487	6.72E-06	--	0.437	1	0.5087	20	40907149
